# The Washing Out Plan—The Good It Fails to Do, the Harm It Does

**Published:** 1903-10

**Authors:** Alexander Haig

**Affiliations:** M. A. and M. D., Oxon, F. R. C. P., London, Physician to the Metropolitan Hospital and to the Royal Hospital for Children and Women


### The Washing Out Plan—The Good It Fails to Do, the Harm It Does.

By ALEXANDER HAIG, M. A. and M. D., Oxon, F. R. C. P., London,

Physician to the Metropolitan Hospital and to the Royal Hospital for Children and Women.

(*Canada Lancet.*)

In old times, 20 or 30 years ago, when nothing was known about the introduction of uric acid, when excessive uric acid was thought to be due to faulty metabolism, and when it was believed that all the uric acid that was swallowed was promptly converted into urea, there was little left for the physician to do but to wash out the excess of uric acid by copious libations of water, combined or not with some alkali; and this he has proceeded to do, more or less, ever since, in spite of the fact which has recently become obvious to not a few, that the plan often fails to do very much good, and sometimes indeed does very much harm. Today, however, we know that the foundation of this pathology is untrustworthy, that all uric acid disease is practically due to swallowing it, and that the administration of large quantities of water does but little to clear it out, and thus fails in the object for which it is given. It has also become evident that in the largest class of uric acid diseases, viz., those of the circulation due to excess of uric acid in the blood (collemia), it not only fails to do the good expected of it, but very often indeed does actually produce infinite harm, even death itself.

And, first, as to its failure to do good.—In my book, Uric Acid, edition 6, page 194, I give notes of an instance in which in a morning hour the excretion of urine was only 53 c.c. per hour, and then a dose of opium taken without fluid ran it up in the next hour to 234 c.c. Here there was obviously plenty of water in the blood, but it was unable to get out till the opium cleared the blood of uric acid, so freeing the renal capillaries and allowing the water to pass. Obviously then, the uric acid in the blood controlled and kept back the water; the water in the blood did not control the uric acid. If a pint of water had been administered that morning before the opium would it have increased the excretion of water? Probably hardly at all, for there was a large amount of uric acid in the blood and it was obviously blocking the renal capillaries and keeping all the water in, and the water was scanty before the opium, not from want of fluid in the body and blood, but from blocking of capillaries by uric acid.

But there is one way in which excess of water may increase the excretion of uric acid, and that is by producing dyspepsia; for dyspepsia diminishes the digestion and absorption of food, and so diminishes the formation of urea and of acid products, and this produces increased alkalinity of the blood and so increased solution of uric acid, and, for a time, increased excretion of uric acid. Probably, at least, one portion of the dyspepsia, produced by the excess of water, is due to its diluting all the digestive fluids and rendering them less active solvents of the food substances submitted to their action. Thus, we may say, to complete this part of the subject, that the uric acid in the blood controls the excretion of water, and that the addition of water to the blood does not control or increase the excretion of uric acid, but that to a certain extent, by diluting the digestive fluids and producing dyspepsia, the continued administration of excess of water does, to a small extent, increase the excretion of uric acid. It is now, however, quite unnecessary to give excess of water at all, for all uric acid disease is prevented by stopping the introduction of uric acid. A person who forms 10 grains of uric acid a day and swallows other 10 grains in his food, will have 20 grains of uric acid to deal with each day, and may suffer more or less severely from uric acid disease; but, if he will take the trouble to shut out the unnecessary 10 grains which he swallows with his food, he will gradually draw clear from excessive uric acid; he will have to deal each day only with 10 grains of the substance, and he will cease to suffer from the severe diseases which its excess in the blood and tissues produces.

But the practice of washing out with excess of fluids, which is due to an antiquated physiology, is still carried on, and it is therefore necessary to show up the harm which it only too often does. In arthritis, due to uric acid, it produces dyspepsia; dyspepsia increases the solvent powers of the blood for uric acid, and so the uric acid is dissolved out of the joints, and collemia, anemia and debility may take the place of the arthritis. The patient is cured of one form of uric acid disease only to suffer, and perhaps, to suffer very severely, indeed, from another form which is, perhaps, worse than the original. That he may get clear of his gout, is true, but this may be replaced by anemia, high blood pressure, with all its intracranial results, with mental depression and other troubles, and the danger of cerebral hemorrhage, or, before long, he may find himself suffering also from Bright's disease, dilated heart and dropsy. But the worst effect of this washing-out plan is seen in the far more numerous diseases which uric acid produces, through its effects on the circulation, those diseases which I have called the collemic group, of which Bright's disease is the worst type. The worst possible thing to happen in all collemic disease and that which makes the prognosis in Bright's disease almost absolutely hopeless, is heart failure, for Bright's disease may be defined as collemia producing defective circulation, the defective circulation being doubled, trebled and quadrupled by subsequent failure of the heart. Collemia itself means defective combustion, but that defective combustion is far more than doubled if the heart fails as well. Now, when the heart is fighting against capillaries obstructed by uric acid, when it has as much as it can do to keep up the circulation from day to day against these obstructed capillaries, it is obvious that a comparatively small matter may turn the scale against it and lead to its dilatation and complete failure. Now, this is exactly what excess of fluid does,—it produces, as we have seen, dyspepsia, and dyspepsia produces defective nutrition, defective nutrition not only throughout the body, but of the most important muscle of all, that of the heart; and then we must not forget that a pint of fluid weighs a pound and a quarter, and that each pint of fluid more in the blood is 1¼ pounds more for the heart to drive. In this way excess of fluid does infinite harm,—it dilates the stomach and causes incurable dyspepsia, dyspepsia causes debility, and the fluid does also increase, to a very large extent, the work that the heart has to do. No wonder the heart fails and dilates; no wonder it ultimately becomes like the bulged outer casing of a bicycle tire, absolutely beyond repair.

Now, in recent years, I have seen quite a number of cases, of which I could give notes did space permit, who have gone through this treatment of washing out and have survived by accident as mere helpless wrecks with hearts which have been stretched beyond endurance and which will never again be fit for good work, lives which are crippled and which must live, if they live at all, on mere sufferance and as invalids. Some of these cases have told me that they were forced to drink pint after pint of water in spite of an absolute distaste and loathing for fluid, (see Uric Acid, Ed. vi., p. 30), for in this case nature says no with all the force at her command. They do not want water, as there is already excess of water in the blood and tissue fluids, but the patients are forced to drink and manage to get down several pints a day in spite of nature's protests and in excess of her requirements. Gradually, between dyspepsia on the one hand, and excess of fluid to drive and excessive obstruction of the capillaries on the other, the heart fails, and dilates, and gives up the struggle, and one day the patients take to fainting severely, or become more or less dropsical, and the worst that can possibly be done for these patients has been accomplished.

Perhaps in the great and extensive employment of water, in the present day of treatments for dilatation and failure of the heart, quite a large number of these cases are probably due to the absolutely unphysiological idea that uric acid can be washed out. It was nearer the truth to say that the patient can be washed out, and is not unfrequently washed out, but the uric acid remains, and, more insane of all, those people who go in for washing out do also introduce each day in the food quite as many grains of uric acid as they could hope to wash out, if the process did act as it is supposed to, by those who have trusted to their imagination and not looked to see what the physiology of the body really does.

If the pericardium is very strong and refuses to give way, then the excess of fluid produces very high blood pressure with headache, mental depression, epistaxis, perhaps cerebral hemorrhage, but in any case the treatment is both painful and dangerous and I have shown that it is useless and unnecessary.

The opposite side of the picture is, I think, to be seen in the enormous amount of good which can be accomplished in many cases of collemia and Bright's disease by cutting off all fluids, by putting the patient on a pint, or even half a pint of fluid in the 24 hours, or even on an absolutely dry diet of bread and fruit, the small amount of fluid in the fresh fruit being all that is taken for days together. The way in which the heart will sometimes recover under these conditions, when not hopelessly damaged, and with the recovery of the heart the way in which the defective combustion which we call Bright's disease will gradually pass away, is an object lesson which many today should witness to show them how extremely silly and unphysiological is the opposite plan of treatment, the attempt to wash out the uric acid from the body in excess of fluid, when the uric acid, as a matter of fact, controls absolutely all output of fluid from the body, and the absolutely insane attempt to cure uric acid disease by sweeping it out with one hand, while pouring it in with the other. In this and all cases of uric acid disease there is no necessity to run such terrible risks, for the uric acid excretion can, in most cases, be reduced to at least one half by cutting off introduction, and if this is done it may be unnecessary to do anything at all to increase its excretion. A somewhat low and dry diet of bread and fruit with, or without, a little alkali, will do easily and safely all that is required.

